# Open fetal surgery for correction of myelomeningocele and umbilical cord presentation at hysterotomy: report of a rare complication

**DOI:** 10.31744/einstein_journal/2026RC1831

**Published:** 2026-06-23

**Authors:** Alex Sandro Rolland Souza, Luanna Vitor Macedo, Gabriela Fonseca Souza, Gustavo Fonseca Albuquerque Souza, Igor Faquini, Orlando Gomes Santos-Neto, Silvia Lourdes Loreto Faquini

**Affiliations:** 1 Instituto de Medicina Integral Prof Fernando Figueira Recife PE Brazil Instituto de Medicina Integral Prof. Fernando Figueira, Recife, PE, Brazil.; 2 Universidade Católica de Pernambuco Recife PE Brazil Universidade Católica de Pernambuco, Recife, PE, Brazil.; 3 Universidade Federal de Pernambuco Recife PE Brazil Universidade Federal de Pernambuco, Recife, PE, Brazil.

**Keywords:** Prenatal care, Prenatal diagnosis, Meningomyelocele, Ultrasonography, Umbilical cord, Hysteretomy

## Abstract

Myelomeningocele is a severe neural tube defect that significantly affects neurological development. Studies have demonstrated that intrauterine repair leads to better neurological outcomes than postnatal correction, likely due to reduced exposure of the neural tissue to amniotic fluid. Despite its benefits, prenatal surgery is performed in only a limited number of centers worldwide and is associated with both maternal and fetal risks. This case report aims to describe a complication rarely reported in the literature: umbilical cord presentation at hysterotomy during open intrauterine fetal surgery for myelomeningocele repair. A 35-year-old multigravida at 24 weeks of gestation was referred to a tertiary maternal-fetal medicine center for evaluation of a suspected myelomeningocele. Ultrasound examination confirmed open spina bifida at the L5 level (2.6 × 2.4cm) associated with lemon and banana signs and a posterior ventricular diameter of 12mm. After counseling, the patient opted for intrauterine repair. During surgery, the umbilical cord was not identified on ultrasound before hysterotomy; however, it was found crossing the hysterotomy site intraoperatively, requiring careful manipulation to achieve adequate fetal exposure. The surgery proceeded without further complications. At 30 weeks of gestation, preterm delivery was indicated due to chorioamnionitis. The newborn had an Apgar score of 8 and 7 and requiring admission to the neonatal intensive care unit. At 1 year and 7 months of age, the child demonstrates good motor development without the need for postnatal surgical interventions. The umbilical cord malpositioning observed during fetal surgery is a rarely described complication in the literature that may compromise fetal circulation; therefore, careful intraoperative handling is essential to prevent adverse outcomes. This case report highlights a rare intraoperative complication of fetal myelomeningocele repair. Appropriate surgical management ensured a favorable outcome, underscoring the importance of awareness and preparedness in managing such events.

## INTRODUCTION

Spina bifida is the most common congenital malformation of the central nervous system and does not pose an immediate threat to life.^(1)^ Myelomeningocele (MMC), resulting from failure of neural tube closure within the first four weeks after fertilization, is the most common form and is characterized by protrusion of a sac containing cerebrospinal fluid, the spinal cord, and nerve roots.^(1)^

Although nonlethal, this malformation is associated with significant morbidity and reduced postnatal quality of life. Since 2011, studies have demonstrated that fetuses with MMC undergoing intrauterine repair have better neurological outcomes than those treated postnatally, likely to reduced exposure of neural tissue to amniotic fluid.^(1)^ Despite the advantages of antenatal correction, only a limited number of centers worldwide perform this procedure, and its potential maternal, intraoperative, and fetal complications must be carefully considered.^(1-3)^

A review of the literature showed that umbilical cord presentation at hysterotomy during open fetal surgery for MMC repair has been rarely reported, despite its potential to result in true cord prolapse and fetal death.^(4,5)^ Given the scarcity of reports on this topic, the present study aimed to describe a case of umbilical cord presentation as a possible complication of open intrauterine fetal surgery for MMC repair.

## CASE REPORT

A 35-year-old multigravid woman at 24 weeks of gestation was referred to the Fetal Medicine Department of *Instituto de Medicina Integral Prof. Fernando Figueira* (Pernambuco, Brazil) in May 2023 due to MMC identified on an obstetric ultrasound performed at an outside institution in another state.

The patient underwent a second-trimester morphological ultrasound, which revealed discontinuity of the lumbosacral spine, confirming the diagnosis of MMC or open spina bifida. The defect originated at the L5 vertebral level and measured approximately 2.6x2.4cm. Additional ultrasound findings associated with this fetal malformation were also observed, including the lemon sign (altered shape of the skull), banana sign (abnormal cerebellar configuration secondary to herniation into the posterior fossa), a posterior ventricular diameter of 12mm, and medial deviation of the right foot.

The patient and her companion received counseling regarding the fetal abnormalities, and the available surgical options, including intrauterine and postnatal repair, were discussed. After explanation of the maternal and fetal risks and benefits, and confirmation that the case met the eligibility criteria proposed by the Management of Myelomeningocele Study (MOMS) - (2011)^(1)^ and endorsed by the Brazilian Federation of Associations of Gynecologists and Obstetricians (FEBRASGO)^(6)^ for intrauterine repair, the couple opted for fetal surgery.

During surgery, the fetal medicine team performed the standard surgical steps according to the modified mini-hysterotomy technique described by Callou et al.^(3)^ These steps included ultrasound-guided identification of the incision site, uterine opening (hysterotomy) measuring approximately 4.5cm to expose the fetus at the site of the MMC, opening of the amniotic membrane with drainage of clear amniotic fluid, and fixation of the amniotic membrane to the uterine wall.^(3)^ Before the uterine incision, intraoperative ultrasound was performed to identify the optimal site for hysterotomy and confirmed the absence of the fetal umbilical cord at the level of the defect.

**Figure 1:**
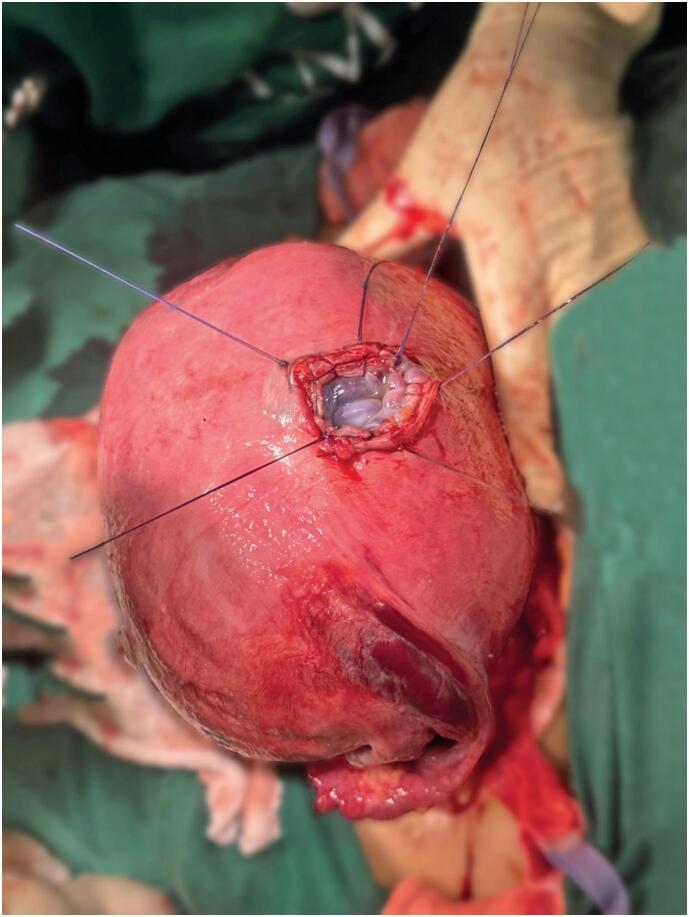
Umbilical cord presentation during hysterotomy for intrauterine myelomeningocele repair

At this stage, under direct visualization, instead of identifying the fetal region affected by MMC, the umbilical cord was observed crossing the hysterotomy site (Figure 1). Careful external manipulation was required to reduce and lateralize the umbilical cord, allowing exposure only of the fetal dorsal region, corresponding to the MMC site. To achieve this, the assistant surgeon inserted the fifth finger between the uterine wall and the fetal back, on the side opposite to the umbilical cord presentation, in order to decompress the cord and facilitate careful manipulation by the primary surgeon. This maneuver was performed without complications. Fetal heart rate (FHR) was monitored throughout the procedure using intraoperative ultrasonography, together with inspection of the umbilical cord. The patient was subsequently transferred to the obstetric intensive care unit (ICU) for postoperative surveillance and was discharged on the fifth postoperative day. During hospitalization after the intervention, fetal follow-up included FHR assessment and cardiotocography. No episodes of fetal bradycardia were observed.

At 30 weeks of gestation, the patient developed signs of chorioamnionitis, and delivery by cesarean section was indicated. The male newborn weighed 1,665g, and had Apgar scores of 8 and 7 at 1 and 5 minutes, respectively. After birth, he was admitted to the neonatal intensive care unit (ICU), where he remained hospitalized for 10 days, followed by an additional 22 days in the ward before hospital discharge. As of February 2025, the child was 1 year and 7 months old demonstrated good motor development, and had not required any postnatal surgical interventions.

### Ethical approval

This study was approved by the Ethics Committee for the Analysis of Research Projects of the *Instituto de Medicina Integral Prof Fernando Figueira* (CAAE: 75980323.3.0000.5201; #6.575.969). Written informed consent was obtained from the patient before the initiation of any research-related procedures.

## DISCUSSION

Neural tube defects are a common congenital malformation that occur around the fourth week of embryogenesis due to failure of embryonic neural tube closure. MMC is the most severe variant compatible with life and the most common form of spina bifida, characterized by the herniation of a sac containing cerebrospinal fluid, the spinal cord, and nerve roots.^(7,8)^

Open fetal surgery for intrauterine MMC repair has been widely reported in the literature, and favorable outcomes were confirmed by the MOMS study, a large randomized controlled trial comparing prenatal and postnatal repair.^(1)^ The classic open fetal surgery for technique MMC correction described in the MOMS trial is performed through a hysterotomy measuring 6.0 to 8.0cm allowing layered repair of the neural tube defect similarly to the postnatal approach.^(1)^ To minimize fetal access and reduce maternal and fetal morbidity, some research groups have investigated less invasive approaches, including endoscopic techniques and open surgery using smaller hysterotomy incisions.^(9,10)^ However, to date, outcomes achieved with endoscopic approaches have not been shown to be superior to those obtained with the open technique, mainly because endoscopic repair limits the spinal cord untethering, a crucial step in MMC correction.^(11)^

The MOMS study demonstrated a statistically significant superiority of intrauterine treatment over postnatal repair regarding several outcomes, including reduced need for ventriculoperitoneal shunt placement, decreased cerebellar herniation at 12 months of age, and greater likelihood of independent ambulation. However, maternal morbidity and gestational complications such as higher rates of premature rupture of membranes, preterm labor, chorioamniotic membrane separation, hysterorrhaphy dehiscence, and the need for maternal blood transfusion at delivery were more frequent following prenatal surgery. The rates of neonatal complications were similar between the two groups, except for a higher incidence of respiratory distress syndrome associated with prematurity in the prenatal surgery group.^(1)^ Despite the complications associated with intrauterine treatment, the study suggested that the benefits of prenatal surgery outweighed the risks.^(8)^

Anomalous exteriorization or presentation of the umbilical cord during open fetal surgery is a rare complication characterized by direct visualization of the cord at the hysterotomy site before fetal exposure. In the present case, rapid and careful external manipulation of the cord was performed to access to the fetal back, by repositioning the cord into the amniotic cavity or lateralizing it to achieve adequate exposure of the fetal surgical field without changes in FHR.

This intraoperative complication may occur during opening of the amniotic cavity or drainage/aspiration of amniotic fluid due to umbilical cord mobility and changes in intrauterine pressure, including after fetal anesthesia. However, few studies have described this event.^(4,5)^ Umbilical cord presentation may also lead to serious complications, such as true cord prolapse, clamping, or compression, potentially compromising fetal blood flow, altering FHR, and increasing perinatal morbidity and mortality.

Throughout the procedure, continuous FHR monitoring was performed, and potential alterations were minimized using a decompression technique in which the assistant surgeon's fifth finger was positioned to relieve pressure on the umbilical cord.

The report of this rare complication was also accompanied by an image showing the umbilical cord at the hysterotomy site (Figure 1) aiming to help other centers recognize and appropriately manage this event through careful manipulating of the umbilical cord.

## Data Availability

The underlying data supporting the findings of this study are contained within the manuscript.
